# Extra Virgin Olive Oil Phenolic Compounds Modulate the Gene Expression of Biomarkers Involved in Fibroblast Proliferation and Differentiation

**DOI:** 10.3390/genes15020173

**Published:** 2024-01-28

**Authors:** Anabel González-Acedo, Rebeca Illescas-Montes, Elvira de Luna-Bertos, Concepción Ruiz, Javier Ramos-Torrecillas, Olga García-Martínez, Lucía Melguizo-Rodríguez

**Affiliations:** 1 Biomedical Group (BIO277), Department of Nursing, Faculty of Health Sciences, University of Granada, C/Santander, 1, 52005 Melilla, Spain; anabelglez@ugr.es; 2 Biomedical Group (BIO277), Department of Nursing, Faculty of Health Sciences, University of Granada, Avda. Ilustración 60, 18016 Granada, Spain; rebecaim@ugr.es (R.I.-M.); elviradlb@ugr.es (E.d.L.-B.); crr@ugr.es (C.R.); jrt@ugr.es (J.R.-T.); luciamr@ugr.es (L.M.-R.); 3 Institute of Biosanitary Research, ibs.Granada, C/Doctor Azpitarte 4, 4ª Planta, 18012 Granada, Spain; 4 Institute of Neuroscience, Centro de Investigación Biomédica (CIBM), University of Granada, Parque de Tecnológico de la Salud (PTS), Avda. del Conocimiento S/N, Armilla, 18016 Granada, Spain

**Keywords:** fibroblast, gene expression, phenolic compounds, olive oil, wound healing

## Abstract

Extra virgin olive oil phenolic compounds have been identified as possible biostimulant agents against different pathological processes, including alterations in healing processes. However, there is little evidence on the molecular mechanisms involved in this process. The aim was to analyse the effect of hydroxytyrosol, tyrosol, and oleocanthal on fibroblast gene expression. PCR was used to determine the expression of different differentiation markers, extracellular matrix elements, and growth factors in cultured human fibroblasts CCD-1064Sk treated with different doses of hydroxytyrosol (10^−5^ M and 10^−6^ M), tyrosol (10^−5^ M and 10^−6^ M), and oleocanthal (10^−6^ M and 10^−7^ M). After 24 h of hydroxytyrosol treatment, increased expression of connective tissue growth factor, fibroblast growth factor (FGF), platelet-derived growth factor, vascular endothelial growth factor, transforming growth factor β1 (TGF-β1), and their receptors was observed. Tyrosol and olecanthal modulated the expression of FGF and TGFβR1. All phytochemicals tested modified the expression of differentiation markers and extracellular matrix elements, increasing gene expression of actin, fibronectin, decorin, collagen I, and III. Phenolic compounds present in extra virgin olive could have a beneficial effect on tissue regeneration by modulating fibroblast physiology.

## 1. Introduction

The integumentary system formed by the skin and its appendages (e.g., hair follicles, nails, sweat, and sebaceous glands) acts as an initial protection barrier against external agents and contributes to maintaining homeostasis [1]. Lesions to these tissues induce a wound healing process that comprises four sequential and predictable phases: hemostasis, inflammation, proliferation, and remodeling [2]. Wound healing is a physiological phenomenon with a signaling network that involves chemokines, growth factors, immune cells, and other cell populations, including endothelial cells, keratinocytes, and fibroblasts [3]. Fibroblasts are the most abundant cell population in the dermis [4]. The role of fibroblasts in maintaining tissue integrity and homeostasis is crucial, as they play an essential role in the wound healing process [4,5]. In the proliferation phase, they are responsible for the breakdown of the fibrin clot and the production of collagen and elastin to form the extracellular matrix (ECM), which is involved in the formation of granulation tissue [6,7]. The ECM plays an important role in various cellular processes, such as cell adhesion, migration, maintenance of cell shape, and proliferation [8]. Fibroblasts actively participate in wound healing from the late inflammatory phase, when they promote re-epithelialization, up to the remodeling phase, when they give rise to a mature scar [6,9]. In this process, various growth factors have an indispensable role, such as transforming growth factor β (TGF-β), platelet-derived growth factor (PDGF), fibroblast growth factor (FGF), vascular endothelial growth factor (VEGF), and interferon γ (IFNγ), which ensure the correct re-epithelialisation and angiogenesis of the tissue [10]. DDR2 collagen receptors play a pivotal role in governing fibroblast proliferation, migration, and extracellular matrix (ECM) synthesis, crucial processes in the context of wound healing. The association between DDR2 and MMP-2, primary proteases in the ECM responsible for wound remodeling, is noteworthy. Consequently, a reduction in DDR2 levels has been observed to diminish fibroblast migration and suppress MMP-2 expression. Within the extracellular matrix (ECM), the proteoglycan decorin assumes critical roles by inactivating both TGF-β and CTGF. This leads to decreased levels of decorin and heightened deposits of elastin fibers in hypertrophic scars when compared to normal skin [11,12,13].

Alterations to this healing process can sometimes lead to the excess formation of scar tissue or to the chronification of the wound [14]. In the former case, keloids can be produced by prolonged inflammation and the abnormal reorganization and remodeling of the collagen fibers that form the ECM [15]. For their part, chronic wounds are characterized by a prolonged inflammatory period, elevated ECM metalloproteinase (MMP) levels, poor tissue oxygenation, increased bacterial load, and decreased growth factor expression [16,17,18]. These abnormalities in wound healing can have a major economic and social impact and impair the quality of life of sufferers. More severe cases, especially of wound chronification, have been closely related to longer hospital stays, more frequent admissions to intensive care units, and higher rates of morbidity and mortality. Hence, there is increasing interest in novel therapies to improve and accelerate wound healing [19,20].

Extra virgin olive oil (EVOO), the main source of fats in the Mediterranean diet, is known to exert a protective effect against cardiovascular diseases [21,22], certain carcinogenic processes [23], and cognitive impairment [24]. EVOO mainly comprises esterified fatty acids alongside other unsaponifiable substances, including phenolic compounds. Various vegetable species contain polyphenols, notably hydroxytyrosol (htyr), tyrosol (tyr), and oleocanthal (ole), bioactive molecules with antioxidant, anti-inflammatory, antimicrobial, and biostimulatory characteristics [25,26]. Phenolic compounds in EVOO may therefore offer an alternative therapeutic approach to multiple pathological processes, including wound healing abnormalities [27]. However, little research has been conducted on how these compounds affect the molecular mechanisms underlying tissue repair. The objective of this study was to determine the effect of EVOO phenolic compounds on the gene expression of fibroblasts, analyzing their growth marker expression and differentiation.

## 2. Materials and Methods

### 2.1. Chemical Products

Commercial standards of htyr, tyr, and ole were obtained from Sigma-Aldrich (St. Louis, MO, USA), dissolved in methanol, and maintained at −20 °C. Pattern solutions were prepared for each substance and used in subsequent solutions. All solvents were analytical or HPLC grade (Sigma-Aldrich), and Milli-Q water was always used (Millipore Corp., Bedford, MA, USA).

### 2.2. Cell Culture

The fibroblast cell line CCD-1064Sk from the American Type Cultures Collection (ATCC, Manassas, VA, USA) was obtained through the Center of Scientific Instrumentation of the University of Granada. Cells were cultured in Dulbecco’s Modified Eagle Medium (DMEM) (Invitrogen Gibco Cell Culture Products, Carlsbad, CA, USA) supplemented with 100 UI/mL penicillin (Lab Roger SA, Barcelona, Spain), 50 μg/mL gentamicin (Braum Medical SA, Jaen, Spain), 2.5 μg/mL amphotericin B (Sigma, St Louis, MO, USA), 1% glutamine (Sigma), 2% HEPES (Sigma), and 10% fetal bovine serum (FBS) (Gibco, Paisley, UK). Cultures were preserved under standard conditions (37 °C, 95% humidity, and 5% CO_2_). Fibroblasts were separated from the culture flask by using 0.05% trypsin (Sigma) and 0.02% ethylenediaminetetraacetic acid (EDTA) (Sigma). Next, cells were washed with phosphate-buffered saline (PBS) and suspended in culture medium with 10% FBS.

### 2.3. RNA Extraction and cDNA Synthesis (Reverse Transcription)

Cells were first incubated for 24 h in the presence of different doses of the phenolic compounds (htyr and tyr: 10^−5^ M and 10^−6^ M; Ole: 10^−6^ M and 10^−7^ M), incubating other cells with culture medium alone as controls. Cells were then separated from the plates using 0.05% trypsin (Sigma, St. Louis, MO, USA) and 0.02% EDTA (Sigma, St. Louis, MO, USA). Next, the protocol described by Manzano-Moreno et al. [28] was followed to extract mRNA from the cells. Briefly, the same amount of RNA (1 μg total RNA in 40 μL of total volume) was reverse-transcribed to cDNA and amplified by polymerase chain reaction (PCR) using the iScript™ cDNA synthesis kit (Bio-Rad Laboratories, Hercules, CA, USA) in accordance with the manufacturer’s instructions. All trials were performed in triplicate.

### 2.4. Real-Time Polymerase Chain Reaction (q-RT-PCR)

Primers based on the NCBI nucleotide library and the Primers design (Table 1) were used to detect mRNA of the following genes: α-actin, collagen I (COL I), collagen III (COL III), connective tissue growth factor (CTGF), discoidin domain receptor 2 (DDR2), decorin, fibroblast growth factor (FGF), fibronectin, matrix metalloproteinase-2 (MMP2), platelet-derived growth factor (PDGF), vascular endothelial growth factor (VEGF), transforming growth factor β1 (TGF-β1) and its receptors (TGFβR1, TGFβR2, and TGFβR3). Results were normalized by using ubiquitin C (UBC), peptidylprolyl isomerase A (PPIA), and ribosomal protein S13 (RPS13) as stable housekeeping genes [29].

The q-RT-PCR technique was performed with the SsoFastTM EvaGreen^®^ Supermix kit (Bio-Rad laboratories) in accordance with the manufacturer’s instructions. Samples were amplified in 96-well microplates in an IQ5-Cycler (Bio-Rad laboratories) at a specific annealing temperature for each gene, ranging from 60 to 65 °C, and at an elongation temperature of 72 °C over 40 cycles. PCR reactions were carried out in a final volume of 20 μL, with 5 μL of cDNA sample and 2 μL of each primer. Ct values were plotted against log cDNA dilution to construct standard curves for each target gene. After each RT-PCR, a melting profile was created, and agarose gel electrophoresis was conducted in each sample to rule out nonspecific PCR products and primer dimers. The comparative Ct method was employed for the relative quantification of gene expression. The mRNA concentration for each gene was expressed as ng of mRNA per average ng of housekeeping mRNAs. The cDNA (≥3 cultures per treatment) from individual cell experiments was determined by q-RT-PCR.

### 2.5. Statistical Analysis

SPSS 26.0 (IBM SPSS, Armonk, NY, USA) was used for statistical analyses. After calculating means and standard deviations, a one-way ANOVA was used to compare the means, setting the standard error at 5%. Dunnett’s post-hoc test was applied for multiple comparisons with controls. The normal distribution of data and variance homogeneity were previously verified using the Shapiro-Wilks and Levene tests, respectively. Results were depicted using Graph-Pad Prism 8 software (La Jolla, CA, USA). Moreover, a molecular interaction network was generated using the GeneMania app to show the relevance of selected genes according to the number of physical and genetic interactions.

## 3. Results

### 3.1. Effect of Phenolic Compounds on the Expression of Fibroblast Growth Factors

**Figure 1:**
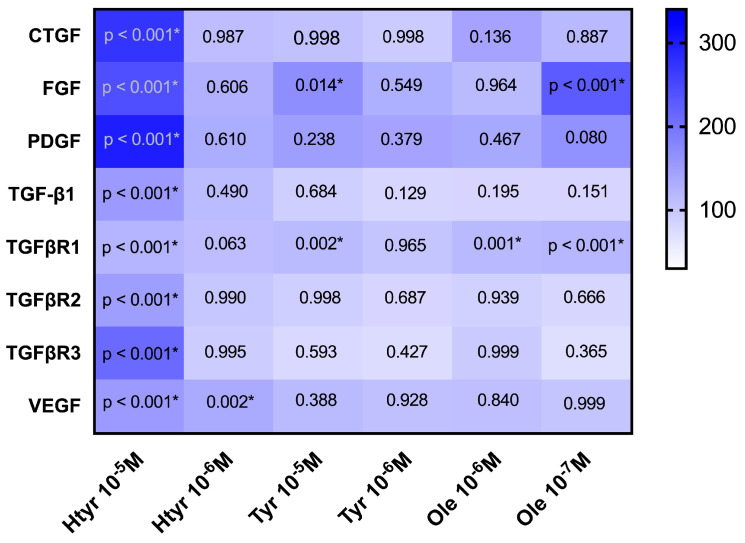
Heat map showing the percentage of gene expression of growth factors in human fibroblasts treated with different doses of htyr, tyr, and ole. The *x*-axis reflects the treatments used, while the *y*-axis reflects the genes studied. All values have been calculated, considering the control as 100%. * represents a *p* value < 0.05 from the ANOVA analysis.

As observed in Table 2, treatment with 10^−5^ M htyr significantly increased (*p <* 0.001) the expression of all growth factors (CTGF, FGF, PDGF, TGF-β1, TGFβR1, TGFβR2, TGFβR3, and VEGF), whereas treatment with 10^−6^ M htyr significantly increased the expression of VEGF alone (*p* = 0.002). Culture with 10^−5^ M tyr significantly increased the expression of FGF (*p* = 0.014) and TGFβR1 (*p* = 0.002). Culture with 10^−6^ M ole significantly increased the expression of FGF (*p* < 0.001), and TGFβR1 expression was increased at both doses tested, 10^−6^ and 10^−7^ M (*p* = 0.001, *p* < 0.001, respectively). Figure 1 represents a heat map showing the percentage of gene expression of growth factors in human fibroblasts treated with different doses of htyr, tyr, and ole. The *x*-axis reflects the treatments used, while the *y*-axis reflects the genes studied.

### 3.2. Effect of Phenolic Compounds on the Expression of Differentiation Markers and Fibroblast ECM Elements

**Figure 2:**
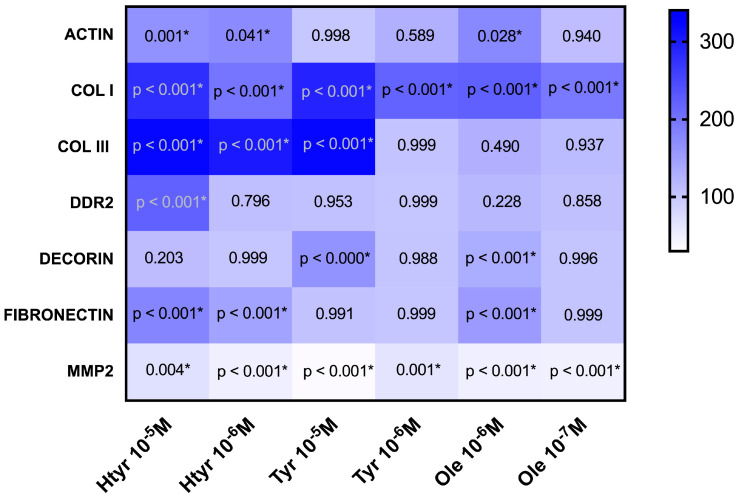
Heat map showing the percentage of gene expression of differentiation markers and extracellular matrix elements in human fibroblasts treated with different doses of htyr, tyr, and ole. The *x*-axis reflects the treatments used, while the *y*-axis reflects the genes studied. All values have been calculated, considering the control as 100%. * represents a *p* value < 0.05 from the ANOVA analysis.

As shown in Figure 2 and Table 3, treatment with 10^−5^ M or 10^−6^ M htyr significantly increased the expression of actin, COL I, COL III, and fibronectin (*p* < 0.05), while treatment with 10^−5^ M htyr increased (*p* < 0.001) the expression of DDR. Treatment with 10^−5^ M htyr increased (*p* < 0.001) the expression of COL I, COL III, and decorin, while treatment with 10^−6^ M tyr increased (*p* < 0.001) the expression of COL I alone. Treatment with 10^−6^ M ole increased (*p* < 0.001) the expression of actin (*p* = 0.028), COL I, decorin, and fibronectin, while treatment with 10^−7^ M increased (*p* < 0.001) the expression of COL I. Both doses of all tested compounds significantly increased the expression of MMP2 (*p* < 0.001).

## 4. Discussion

**Figure 3:**
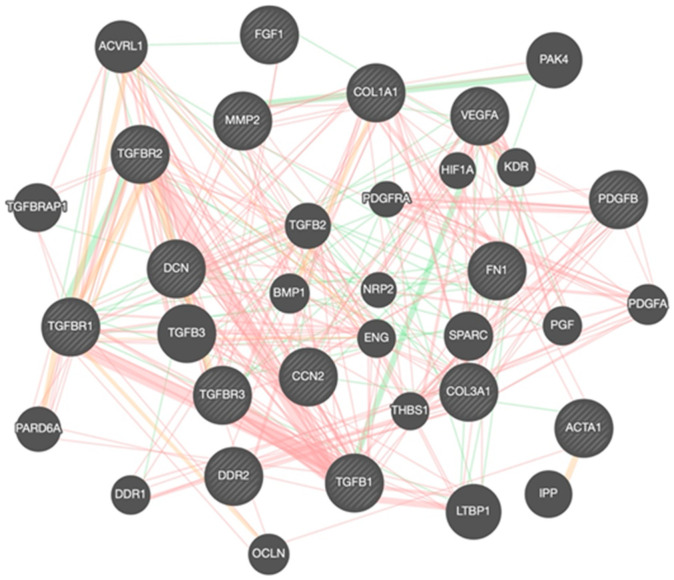
Molecular network representation composed of 15 genes selected for this study (circles with lines inside them) and another 20 closely linked genes (smooth circles). The red lines represent physical interactions, the green lines indicate genetic interactions, and the orange lines show the prediction between the genes.

The main finding of this in vitro study was that treatment with htyr, tyr, or ole, phenolic compounds present in EVOO, may increase the expression of genes involved in tissue repair, including growth factors, differentiation markers, and ECM elements (Figure 3). However, it must be taken into account that the correlation between gene expression and mRNA levels is not always direct, due to different factors such as post-transcriptional modifications or mRNA stability [30]. In any case, the findings are of special relevance because downregulation of these genes may be responsible for delaying wound healing.

Specifically, all doses of the tested compounds upregulated the expression of FGF, which contributes to wound closure by increasing granulation tissue production and promoting re-epithelialization and remodeling [31,32]. Treatment with 10^−5^ M htyr upregulated the expressions of PDGF and DDR2, which are associated with cell proliferation, promoting fibroblast growth, and the consequent increase in ECM production [11,33]. These results are in agreement with previous reports of increased cell proliferation and migration in human fibroblasts treated with EVOO compounds [25]. In the same line, the expression of PDGF, FGF, and TGF-β1 was found to be upregulated by the treatment of cultured human fibroblasts and gastric epithelial cells with other phenolic compounds present in EVOO (caffeic, p-coumaric, and ferulic acids) or with rosmarinic acid, a derivative of caffeic acid in plants of the Laminaceae family. These healing effects have been related to changes in the apoptosis, proliferation, survival, and phosphorylation of proteins such as extracellular signal-regulated kinases (ERK) 1/2 and p38 mitogen-activated protein kinase (MAPK) [34,35].

VEGF and different ECM elements participate in the complex process of angiogenesis by favoring the formation of the new vessels required to transport oxygen, carbon dioxide, and metabolites for tissue regeneration and complete wound closure [36]. In the present study, the expression of VEGF was increased by treatment with htyr at doses of 10^−5^ M and 10^−6^ M. In this context, diabetic foot ulcers were successfully treated using nanoparticles of sesamol [3, 4-methylenedioxyphenol], a natural organic compound obtained from sesame oil, which achieved an acceleration of wound healing mediated by the co-expression of PDGF and VEGF [37]. It has been observed that treatment with 10^−5^ M htyr upregulates the expression of CTGF, which exerts chemotactic and mitogen activity in cells that form connective tissue, helping to synchronize a combined cell response to the lesion [38]. Expression of this gene is often conditioned by the expression of TGF-β1 [39], and treatment with htyr has been found to upregulate the expression of TGF-β1 and its receptors, suggesting a possible increase in the proliferative capacity of fibroblasts and their subsequent differentiation into myofibroblasts, which are responsible for wound contraction [40]. Saika et al. attributed TGF-β1 expression with a key role in wound healing mediated by protein Smad7, an important signaling inhibitor of the TFG-B family; in this way, the interaction of Smad7 with TGF-β R1 blocks the phosphorylation and activation of Smads restricted to the receptor, preventing excessive signal propagation and the formation of hypertrophic scars [41]. Treatment with other bioactive compounds such as curcumin, in combination with chitosan and collagen, was found to modulate the expression of TGF-β1 and its antagonist Smad7 in a punch wound model using male Wistar rats [42,43].

In the present study, treatment with 10^−5^ M tyr or 10^−6^ M ole upregulated the expression of decorin, a proteoglycan that regulates the ECM by inhibiting the expression of TGF-β1 and CTGF. Elevated TGF-β1 and CTGF levels are useful to produce new cicatricial tissue, but they must be regulated to avoid the emergence of keloids. In this regard, the findings on the expression of decorin, TGF-β1, and CTGF may evidence the onset of a regulatory process designed to prevent keloid formation [12,44]. The emergence of keloids and chronification of wounds has been associated with a prolonged increase in the expression of MMPs [45], which are also involved in ECM remodeling [46]. In the present study, MMP2 expression was significantly downregulated after 24 h of treatment with all doses of tested compounds [47].

The expression of COL I and COL III was upregulated after treatment with each compound at each dose. Treatment of fibroblasts with soybean and zein corn proteins has demonstrated a similar effect on fibroblasts, increasing the expression of COL II and 7, integrin-α2, and laminin-β3, among others. The different structural forms of collagen maintain the mechanical resistance and elasticity of the skin and act as substrates for cell proliferation and differentiation [48]. The increased synthesis of collagen has therefore become a therapeutic target of interest to treat skin lesions [49]. Increased expressions of actin and fibronectin were observed in cultures treated with 10^−5^ M or 10^−6^ M htyr or with 10^−6^ M ole, and these markers induce the differentiation of fibroblasts into myofibroblasts, which participate in tissue inflammation, repair, and remodeling [50,51].

Phenolic compounds derived from plant sources exhibit various beneficial effects on human health, which are attributed to their antioxidant, anti-inflammatory, anti-tumor, and other properties. The manifestation of these effects is strongly linked to the bioavailability of these compounds in the body. The bioaccessibility, and therefore the bioavailability, of phenolic compounds is strongly influenced by their structure and the way in which they are introduced into the body. Additionally, the interaction of phenolic compounds with each other or with other macromolecules present in food or during digestion, such as proteins, lipids, dietary fibers, and polysaccharides, has a significant impact on their bioaccessibility and therefore on the desired effect. However, due to the complexity of the mechanisms through which phenolic compounds act in the body, this area has not yet been fully explored, which may represent a limitation of the present work. However, several in vivo studies have shown that direct administration of olive oil to different types of wounds could improve wound healing processes. In this regard, Nassiri et al. (2015) found that EVOO applied to diabetic foot ulcers was effective, improving the complete healing of the ulcer and significantly reducing its size and depth without adverse effects [52]. Similarly, isolated administration of phenolic compounds present in EVOO has also been shown to be useful in animal studies. Bairagi et al. (2018) observed that topical administration of ferulic acid improved skin wound closure by decreasing epithelialization time [53].

Also, oleuropein, applied to the injured skin of male Balb/c mice, decreased cellular infiltration at wound sites, elevated collagen fiber formation, and accelerated re-epithelialization, probably due to the upregulation of VEGF protein [54].

However, it would be interesting for future research to develop tests to simulate gastrointestinal digestion to evaluate in depth the bioaccessibility of the phenolic compounds in EVOO or to explore new encapsulation systems that allow targeted administration to a specific tissue to ensure the controlled release of the dose to be administered [55].

In summary, bioactive compounds in various vegetable species may represent an alternative option to treat chronic wounds or keloids. However, it should be noted that the benefits of polyphenols described in this study are limited to the conditions recreated in the manuscript and the doses used, which could represent a limitation of the present study. In this sense, it would be desirable to consider further research that takes into account other circumstances inherent to wounds with a torpid evolution, such as an inflammatory environment or the presence of contamination. In the same vein, it would also be necessary to develop new studies that examine the responses of other cell populations to these compounds and validate the present findings in more complex in vivo models, studying the safety of their application.

## 5. Conclusions

All phytochemicals tested modified the expression of differentiation markers and extracellular matrix elements, increasing gene expression of actin, fibronectin, decorin, collagen I, and III. Phenolic compounds present in extra virgin olive could have a beneficial effect on tissue regeneration by modulating fibroblast physiology. These findings demonstrate that the phenolic compounds present in EVOO modulate the expression of genes involved in tissue regeneration in cultured human fibroblasts. These bioactive compounds may therefore be candidates for inclusion in care protocols for wounds with torpid development by functionalization of dressings or hydrogels, although further studies are required to evaluate their potential application in the clinical setting.

## Figures and Tables

**Table 1:** Target gene primer sequences for the amplification of cDNA by q-RT-PCR.

Gene	Sense Primer	Antisense Primer	Amplicon (bp)
*α-ACTIN*	5′-TCCTGCTCCTCTCTGTCTCAT-3′	5′-AGTCAGAGCTTTGGCTAGGAA-3′	96
*COL I*	5′-CCTCATCGCAGGAGAAAAAG-3′	5′-CCCTGAAGTGACTGGGGTAA-3′	169
*COL III*	5′-CTACTTCTCGCTCTGCTTCAT-3′	5′-CACCACCTTCACCCTTATCTC-3′	373
*CTGF*	5′-CCTGGTCCAGACCACAGAGT-3′	5′-TGGAGATTTTGGGAGTACGG-3′	194
*DDR2*	5′-GAACCCAAACATCATCCATC-3′	5′-CTTCATGCCAGAGGCAATTT-3′	199
*DECORIN*	5′-GGGCTGGCAGAGCATAAGTA-3′	5′-CAGAGCGCACGTAGACACAT-3′	196
*FGF*	5′-CCCATATTCCCTGCACTTTG-3′	5′-ACCTTGACCTCTCAGCCTCA-3′	195
*FIBRONECTIN*	5′-GCCATGACAATGGTGTGAAC-3′	5′-GCAAATGGCACCGAGATATT-3′	200
*MMP2*	5′-CCAAGAACTTCCGTCTGTCC-3′	5′-TGAACCGGTCCTTGAAGAAG-3′	195
*PDGF*	5′-AGATCGAGATTGTGCGGAA-3′	5′-CTTGTCATGCGTGTGCTT-3′	720
*TGF-β1*	5′-TGAACCGGCCTTTCCTGCTTCTCATG-3′	5′-GCGGAAGTCAATGTACAGCTGCCGC-3′	152
*TGFβR1*	5′-ACTGGCAGCTGTCATTGCTGGACCAG-3′	5′-CTGAGCCAGAACCTGACGTTGTCATATCA-3′	201
*TGFβR2*	5′-GGCTCAACCACCAGGGCATCCAGATGCT-3′	5′-CTCCCCGAGAGCCTGTCCAGATGCT-3′	139
*TGFβR3*	5′-ACCGTGATGGGCATTGCGTTTCCA-3′	5′-GTGCTCTGCGTGCTGCCGATGCTGT-3′	173
*VEGF*	5′-CCTTGCTGCTCTACCTCCAC-3′	5′-CACACAGGATGGCTTGAAGA-3′	197

Abbreviations: COL I, collagen 1; COL III, collagen 3; CTGF, connective tissue growth factor; DDR2, discoidin domain receptor 2; FGF, fibroblast growth factor; MMP2, matrix metalloproteinase-2; PDGF, platelet-derived growth factor; TGF-β1, transforming growth factor β1; TGFβR1, transforming growth factor β receptor 1; TGFβR2, transforming growth factor β receptor 2; TGFβR3, transforming growth factor β receptor 3; VEGF, vascular endothelial growth factor.

**Table 2:** Effect of phenolic compounds on the expression of fibroblast growth factors. Data are expressed as Mean ± SD of ng of mRNA per average ng of housekeeping mRNAs.

Gene	Treatment	Mean	S.D.	M.D.
*CTGF*	Control	467.02	17.60	-
	Htyr 10^−5^ M	1273.45	205.14	806.43
	Htyr 10^−6^ M	510.29	26.01	43.28
	Tyr 10^−5^ M	497.70	146.89	30.68
	Tyr 10^−6^ M	437.74	71.03	−29.28
	Ole 10^−6^ M	660.00	14.60	192.99
	Ole 10^−7^ M	538.09	23.20	71.08
	Ole 10^−7^ M	1172.11	142.38	26.22
*FGF*	Control	1.17	0.14	-
	Htyr 10^−5^ M	2.83	0.12	1.66
	Htyr 10^−6^ M	1.49	0.28	0.32
	Tyr 10^−5^ M	1.97	0.53	0.80
	Tyr 10^−6^ M	1.51	0.37	0.34
	Ole 10^−6^ M	1.33	0.28	0.16
	Ole 10^−7^ M	2.66	0.87	1.49
*PDGF*	Control	20.17	3.21	-
	Htyr 10^−5^ M	61.07	11.60	40.56
	Htyr 10^−6^ M	27.19	9.20	6.68
	Tyr 10^−5^ M	30.56	13.42	10.05
	Tyr 10^−6^ M	29.06	12.50	8.55
	Ole 10^−6^ M	28.30	5.11	7.79
	Ole 10^−7^ M	33.51	3.27	13.00
*TGF-β1*	Control	134.77	2.47	-
	Htyr 10^−5^ M	206.80	25.95	72.03
	Htyr 10^−6^ M	152.10	38.80	17.33
	Tyr 10^−5^ M	120.69	12.68	−14.08
	Tyr 10^−6^ M	108.36	5.77	−26.41
	Ole 10^−6^ M	110.84	18.98	−23.93
	Ole 10^−7^ M	109.29	11.38	−25.49
*TGFβR1*	Control	30.31	1.45	-
	Htyr 10^−5^ M	37.40	1.38	7.09
	Htyr 10^−6^ M	33.27	4.12	2.96
	Tyr 10^−5^ M	34.87	1.18	4.56
	Tyr 10^−6^ M	31.07	1.18	0.75
	Ole 10^−6^ M	35.14	1.44	4.82
	Ole 10^−7^ M	36.01	1.17	5.70
*TGFβR2*	Control	64.90	8.42	-
	Htyr 10^−5^ M	103.20	11.96	33.96
	Htyr 10^−6^ M	68.60	11.65	−0.64
	Tyr 10^−5^ M	62.33	13.06	−6.91
	Tyr 10^−6^ M	56.27	14.29	−12.97
	Ole 10^−6^ M	59.50	11.64	−9.74
	Ole 10^−7^ M	56.05	14.98	−13.19
*TGFβR3*	Control	15.62	2.02	-
	Htyr 10^−5^ M	32.92	9.99	17.30
	Htyr 10^−6^ M	14.42	1.49	−1.21
	Tyr 10^−5^ M	12.03	2.69	−3.59
	Tyr 10^−6^ M	11.37	4.06	−4.25
	Ole 10^−6^ M	14.90	4.44	−0.72
	Ole 10^−7^ M	11.09	1.58	−4.54
*VEGF*	Control	11.49	1.60	-
	Htyr 10^−5^ M	17.65	1.75	6.16
	Htyr 10^−6^ M	15.52	2.41	4.03
	Tyr 10^−5^ M	13.14	1.70	1.64
	Tyr 10^−6^ M	12.28	1.57	0.78
	Ole 10^−6^ M	12.46	1.58	0.96
	Ole 10^−7^ M	11.72	1.28	0.22

S.D., Standard Deviation; M.D., Mean differences.

**Table 3:** Effect of phenolic compounds on the expression of differentiation markers and fibroblast ECM elements. Data are expressed as Mean ± SD of ng of mRNA per average ng of housekeeping mRNAs.

Gene	Treatment	Mean	S.D.	M.D.
*ACTIN*	Control	0.07	0.03	-
	Htyr 10^−5^ M	0.19	0.09	0.06
	Htyr 10^−6^ M	0.15	0.02	0.06
	Tyr 10^−5^ M	0.08	0.03	−0.01
	Tyr 10^−6^ M	0.11	0.04	0.02
	Ole 10^−6^ M	0.15	0.02	0.07
	Ole 10^−7^ M	0.09	0.04	0.01
*COL I*	Control	583.28	21.51	-
	Htyr 10^−5^ M	1623.46	33.48	1040.18
	Htyr 10^−6^ M	1158.62	33.20	575.34
	Tyr 10^−5^ M	1698.38	47.03	1115.09
	Tyr 10^−6^ M	1276.28	49.75	692.99
	Ole 10^−6^ M	1302.28	24.47	718.99
	Ole 10^−7^ M	1132.27	40.16	548.98
*COL III*	Control	0.35	0.06	-
	Htyr 10^−5^ M	1.15	0.10	0.79
	Htyr 10^−6^ M	1.07	0.10	0.72
	Tyr 10^−5^ M	1.15	0.07	0.80
	Tyr 10^−6^ M	0.36	0.03	0.01
	Ole 10^−6^ M	0.44	0.001?	0.09
	Ole 10^−7^ M	0.38	0.08	0.05
*DDR2*	Control	29.16	3.99	-
	Htyr 10^−5^ M	64.63	2.33	35.47
	Htyr 10^−6^ M	31.67	1.84	2.52
	Tyr 10^−5^ M	30.84	2.54	1.68
	Tyr 10^−6^ M	34.12	3.28	0.43
	Ole 10^−6^ M	34.93	4.12	4.96
	Ole 10^−7^ M	31.41	2.19	2.25
*DECORIN*	Control	1145.88	29.63	-
	Htyr 10^−5^ M	1279.36	18.99	133.48
	Htyr 10^−6^ M	1159.38	41.10	13.50
	Tyr 10^−5^ M	1857.01	52.13	711.13
	Tyr 10^−6^ M	1178.76	123.95	32.88
	Ole 10^−6^ M	1519.44	24.05	373.56
	Ole 10^−7^ M	1172.11	142.38	26.22
*FIBRONECTIN*	Control	2258.37	117.32	-
	Htyr 10^−5^ M	4032.37	420.24	1773.99
	Htyr 10^−6^ M	3251.06	455.03	992.69
	Tyr 10^−5^ M	2368.10	296.41	109.73
	Tyr 10^−6^ M	2295.70	231.03	37.33
	Ole 10^−6^ M	3436.54	639.48	1178.17
	Ole 10^−7^ M	2301.49	240.47	43.12
*MMP2*	Control	478.61	40.81	-
	Htyr 10^−5^ M	327.64	38.62	−150.97
	Htyr 10^−6^ M	237.91	14.74	−240.70
	Tyr 10^−5^ M	165.70	59.46	−312.91
	Tyr 10^−6^ M	305.00	67.09	−173.61
	Ole 10^−6^ M	236.85	2.76	−241.77
	Ole 10^−7^ M	220.88	46.72	−257.74

S.D., Standard Deviation; M.D., Mean differences.

## Data Availability

Dates are contained within the article.
